# Synergistic Effects of Nitrosamine-Safe Accelerators for Enhanced Natural Rubber Latex Balloon in Sulfur Conventional Vulcanizing System

**DOI:** 10.3390/polym18040438

**Published:** 2026-02-09

**Authors:** Thanakan Chang-In, Ekasit Anancharoenwong, Sunisa Suchat

**Affiliations:** Faculty of Science and Industrial Technology, Prince of Songkla University, Surat Thani Campus, Surat Thani 84000, Thailand

**Keywords:** natural rubber latex, nitrosamine-safe, synergistic accelerator system, mechanical properties

## Abstract

The vulcanization of natural rubber latex (NRL) relies on accelerators to achieve effective crosslinking by a sulfur conventional vulcanizing system and desirable properties such as tensile strength and elasticity. This study investigates the synergistic effects of carcinogenic nitrosamine-safe accelerators to develop a high-performance and safe NRL vulcanization system. A synergistic combination of 0.36 phr Zinc dibutyldithiocarbamate (ZBEC), 0.36 phr Tetrabenzylthiuram disulfide (TBzTD), and a trace amount of Zinc diethyldithiocarbamate (ZDEC), 0.03 phr, demonstrated optimal performance, yielding superior tensile strength (22.13 MPa), elongation at break (1153%), and thermal stability (Tmax 384.15 °C). Notably, this formulation exhibited below the detectable level limits of hazardous nitrosamines (*N*-nitrosodimethylamine (NDMA) and *N*-nitrosodibutylamine (NDBA)). The synergistic nitrosamine-safe accelerator system offers a promising strategy for producing environmentally responsible and consumer-safe NRL products with enhanced mechanical and thermal properties.

## 1. Introduction

The global balloon production volume is valued at around USD 1.85 billion in 2024, with growth expected to reach USD 3.38 billion by 2033, securing a significant market size and a consistent production volume increase across the world [1,2]. However, the production volume of natural rubber balloons is a small percentage of the total natural rubber production, as the global production of natural rubber in 2024 amounted to 28.8 million tons. Rubber balloons are thin gauge rubber products produced from natural rubber latex (NRL) compounds [3]. NRL balloons have superior elasticity, being capable of stretching to seven to eight times their original length while maintaining resilience: a quality not reached by synthetics. This exceptional performance has established rubber balloons as one of the most affordable and universally popular toys across all demographic segments [4]. Beyond children’s entertainment, these versatile products serve multiple functions including celebratory decorations, retail environment enhancement, target practice activities, and festive displays, contributing to their sustained market growth.

However, this seemingly innocuous product harbors a concerning health risk: natural latex can break down to form nitrosamines, a group of known carcinogens detected in various latex products (rubber nipples and gloves) [5,6]. This discovery has prompted the development of methods for extraction and concentration of volatile nitrosamine from latex balloons, highlighting an urgent need to address safety concerns in this growing industry without compromising the product’s widespread appeal and accessibility.

Most toxicological assessments of nitrosamines focus on their carcinogenic and mutagenic properties. Nitrosatable substances can be converted into carcinogenic nitrosamines through reactions with nitrosating agents such as nitrite and nitrogen oxides [7]. Endogenous formation of *N*-nitroso compounds occurs via nitrosation (by nitrite derived from nitrate) of ureas, guanidine, amides, amino acids, and (primary, secondary and aromatic) amines [8]. Of the over 300 different nitrosamines, RIVM (2003) [9] identified *N*-nitrosodimethylamine (NDMA) and *N*-nitrosodibutylamine (NDBA) as being the most prevalent ones, being detected in 96% and 61% of samples, respectively. Both are classified by IARC and the EU as category 2 carcinogens [10]. NDMA, with the generic chemical structure R_2_N-N=O, is the most frequently detected carcinogenic nitrosamine, another form of nitrosamines, although it is of a less dangerous class (BAuA). The European Union has established specific limits for nitrosamines and nitrosatable substances in natural rubber balloons according to European standard EN71-12 [11,12]. The Federal Republic of Germany has proposed limits for nitrosamines and nitrosatable substances in balloons, which are 50 µg/kg and 1000 µg/kg, respectively. Traditionally, nitrosamines and nitrosatable substances are analyzed using thermal energy analyzers (TEA), but due to the limited availability of this expensive equipment type, an improved analysis method using the more widely available gas chromatography–mass spectrometry (GC-MS) technique has been developed based on the EN 12868:2017 standard [13,14,15].

Natural rubber latex balloons can release carcinogenic nitrosamines when certain vulcanization accelerators (carbamates such as zinc diethyldithiocarbamate; ZDEC) are used during balloon production [16]. These substances pose health risks when released during balloon inflation or mouthing, with children being particularly vulnerable. Research by Smith and Norris (2003) [17] identified hazardous exposure levels when children mouth balloons with high nitrosamine content. The primary problematic accelerators are ZDEC, which shows mutagenic activity in bacterial tests, and tetramethyl thiuram disulfide (TMTD), which is a suspected carcinogen: an avoidable threat [18]. Fortunately, safe alternatives exist, namely zinc n-butylbenzothiazole-2-sulfenamide (ZBEC) and tetrabenzylthiuram disulfide (TBzTD) [19]. These alternatives contain low nitrosatable substances, effectively eliminating nitrosamine formation while maintaining product quality. The rubber industry has the knowledge and the technology to produce safer balloon products by transitioning to these alternative accelerators, making this health risk entirely preventable.

This study aimed to comprehensively investigate the performance characteristics of a nitrosamine-safe binary accelerator system comprising TBzTD and ZBEC in the sulfur vulcanization of an NRL balloon. To evaluate the cure characteristics, the swelling index for dipping processability and chemical control were tested for NRL compounds containing various ratios of ZBEC and TBzTD accelerators. The mechanical properties (modulus, elongation at break and tensile strength) of vulcanizates before and after aging, FTIR of the functional groups and the crosslinks formed when prepared with different combinations of the TBzTD/ZBEC accelerator system were determined. The potential synergistic effects that occur when progressively replacing ZDEC with combinations of TBzTD and ZBEC accelerators were assessed. The synergistic nitrosamine-safe accelerator systems are essential to ensure that rubber products (such as balloons), achieve the required mechanical properties while fully complying with stringent safety standards concerning nitrosamine content.

## 2. Experimental

This research was divided into five steps, beginning with optimizing the formulation to be devoid of nitrosamine-producing substances, as follows.

### 2.1. Materials

(1) Materials in NRL films

The centrifuged concentrated latex (Hevea brasiliensis), as a high-ammonia latex (HA) with 60% dry rubber content (DRC), was supplied by Muang Mai Guthrie Public Company Limited, Thailand. The NRL compound formulations for balloon rubber products (Table 1 and Table 2) were purchased from Thanodom Technology Co., Ltd., Bangkok, Thailand. Cellulose nanocrystals (CNC) were obtained as a white powder from rubber seed shells. The average molecular weight was 14,700 g/mol, with particle lengths ranging from 100 to 300 nm and diameters of 20 nm. A 5% CNC dispersion was prepared at a low filler loading of 2 phr.

### 2.2. Preparation of NRL Films

**Figure 1:**
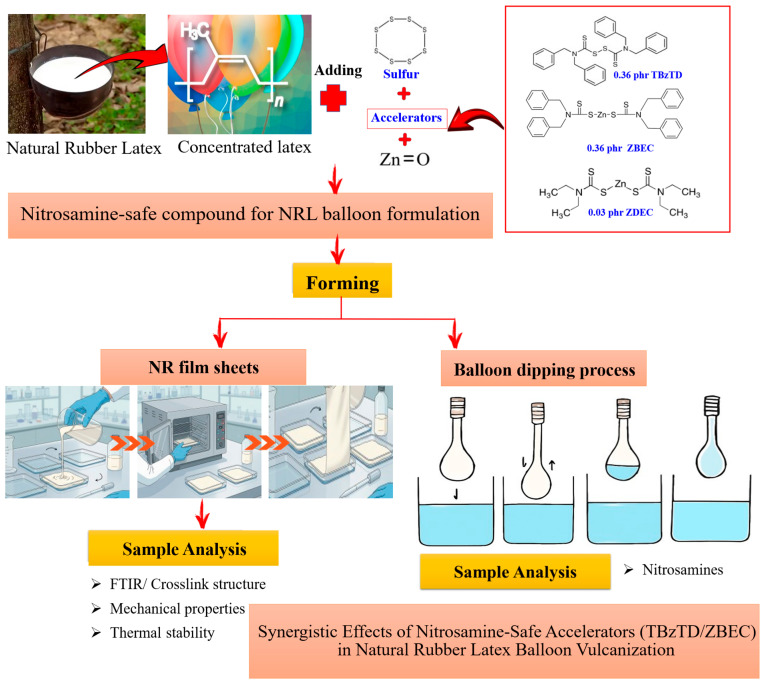
Experimental scheme to test synergistic activities in sulfur vulcanization of NRL balloon products.

The NRL balloon compound formulation combinations of the TBzTD/ZBEC accelerator system were optimized to reduce the tested generation of nitrosamines. Specifically, concentrated latex was used with various types and volumes of accelerators, such as TBzTD, ZDEC, and ZBEC (singly), as well as combinations like TBzTD/ZBEC (binary) and TBzTD/ZBEC/ZDEC (ternary) in NRL. These choices were made to prevent carcinogenic nitrosamine formation. The NRL compound was prepared as a liquid mixture with additives, and mixed in a blender at 2 rpm for 20 min at room temperature. The final mixture was degassed to remove the entrapped air and then poured into glass molds, with eventual film thickness control by the total solid content. The films were cured at 80 °C for 24 h to evaporate water and achieve uniform films. The dried films were peeled off the molds and stored for characterization. By adjusting the types and amounts of accelerators and controlling parameters such as 62% total solid content and a 103% swelling index, the synergistic activities in sulfur vulcanization of NRL films formulations were assessed, emphasizing safety and mechanical properties. A diagram of the experimental design is shown in Figure 1.

### 2.3. NRL Balloon

The NRL balloon dipping process used a balloon mold, clean and dry, soaked in 10% calcium chloride coagulant and slightly dried at 45 °C, and then dipped in the NRL compound. The soaking time was 18 s, and to reuse the mold, it was washed with hot water at 70 °C for 10 min to remove coagulant or residues. The vulcanization dries the balloon in an oven at 100 °C for 1 h to complete the vulcanization process. Samples were removed from the oven and allowed to cool to room temperature. Talcum powder was applied to the balloon surface to prevent sticking.

The finished balloons were carefully removed from the mold, and samples without flaws or damage were kept for the nitrosamine test using GC-TEA, following the standard procedure based on EN 12868:2017 [20].

### 2.4. Characterization and Properties

#### 2.4.1. The Swelling Test

The development of the NRL compound formula from concentrated latex involved varying the types and amounts of accelerator agents. In a sulfur vulcanized system, a stabilizer, activators, and an antioxidant were added to the NRL, and the mixture was matured until a swelling index of 103% was reached, indicating partial crosslinking via sulfur bridges. The compound was then poured onto a 2 mm thick layer on a glass plate, dried at 120 °C for 30 min, and finally cured by heating. The swelling test followed ISO 124, using 3.0 cm circular samples. The diameter was measured carefully at three points and the average was recorded, after which the specimens were immersed in toluene at 23 ± 2 °C for 3 h. The swelling (%) was calculated from the change in diameter caused by immersion, and the average is reported. The percentage swelling was computed as follows.

% Swelling = [(B − A)/A] × 100 (1)

where A is the initial diameter of the specimen before immersion in toluene, and B is the diameter of the specimen after immersion in toluene for 3 h.

For reliable results, this test was run in triplicates and the mean value was recorded, along with test conditions and sample identification.

#### 2.4.2. FTIR Analysis

Fourier transform infrared spectroscopy (FTIR) was employed in transmission mode to analyze the structure, using a Spectrum Two FT-IR spectrometer equipped with a DTGS detector, manufactured by PerkinElmer, Inc., Waltham, MA, USA. Thin rubber sheets with smooth surfaces were analyzed in transmission mode, utilizing the attenuated total reflectance (ATR) accessory for solid-state sampling. FTIR spectra were recorded across the broad wavenumber range from 4000 to 400 cm^−1^ at a resolution of 4 cm^−1^.

#### 2.4.3. Mechanical Properties

The mechanical properties of the NRL film samples are reported as averages of five replicates. Tensile properties were measured, determining the modulus, strength and elongation at break, following the ASTM D 412 standard using a Tinius Olsen 10ST tensile testing machine (Honey Crock Lane, UK). Dumbbell Type C specimens were tested at a speed of 500 mm/min with a 5 kN load cell.

#### 2.4.4. Thermal Stability

(1) Accelerated aging was conducted following ISO 188, where samples were subjected to thermal aging at 70 °C for 168 h. After aging, tensile strength, elongation at break, and modulus were tested.
(2) Thermogravimetric analysis (TGA) was performed using a TGA-SDTA 851 analyzer (Mettler Toledo, Zurich, Switzerland). Samples were heated from 30 °C to 600 °C under nitrogen atmosphere, then from 600 °C to 900 °C under oxygen atmosphere, both at a 10 °C/min heating rate.

#### 2.4.5. Nitrosamines and Nitrosatable Substances

Nitrosamine testing of the balloon latex compound was done using a Gas Chromatography–Thermal Energy Analyzer (GC-TEA) at TÜV Rheinland Hong Kong Ltd., Tsuen Wan, Hong Kong. Nitrite migration from the samples was assessed following the European standard procedure, based on EN 12868:2017 [20]. This method determines nitrosamines by first extracting them into artificial saliva at 40 °C to simulate migration. Nitrosamines require an acidification step for conversion prior to extraction and concentration. The prepared extracts are quantified using GC-TEA, due to its high specificity for nitroso compounds. The reported results are compared to standard limits (migratable nitrosamines, 0.05 mg/kg, and migratable nitrosatable substances, 1.0 mg/kg). The twelve target *N*-nitrosamines identified are *N*-nitrosodimethylamine (NDMA), *N*-nitrosodiethylamine (NDEA), *N*-nitrosodipropylamine (NDPA), *N*-nitrosodibutylamine (NDBA), *N*-nitrosodiisobutylamine (NDiBA), *N*-nitrosoethylphenylamine (NEPhA), *N*-nitrosopiperidine (NPIP), *N*-nitrosopyrrolidine (NPYR), *N*-nitrosomorpholine (NMOR), *N*-nitrosomethylphenylamine (NMPhA), *N*-nitrosodiisononylamine (NDiNA), and *N*-nitrosodibenzylamine (NDBzA).

## 3. Results and Discussion

### 3.1. Characterization and Properties

#### 3.1.1. The Swelling Test

**Figure 2:**
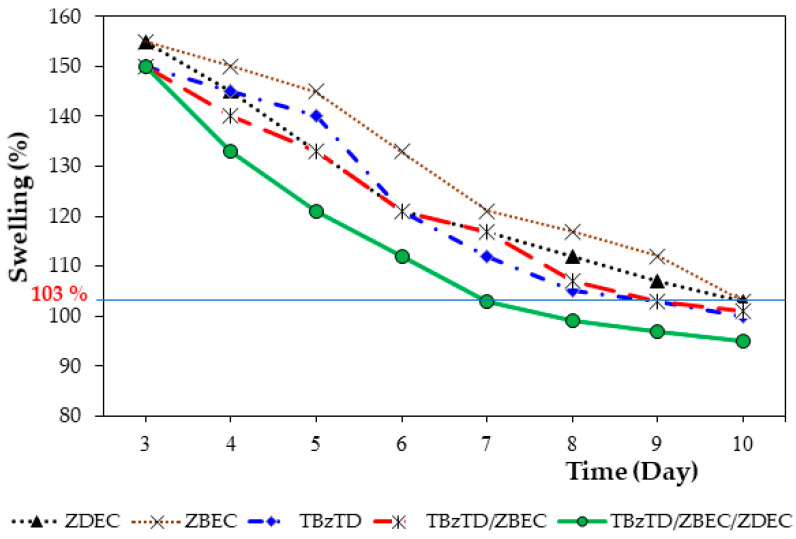
Swelling results are indicative of crosslink bond formation of compound rubbers with different accelerators.

Figure 2 shows the results that are indicative of the crosslinking efficiency of individual accelerators (all tested) over time, with the swelling in all cases decreasing with the curing time, confirming continuous crosslink formation. The nitrosamine-safe alternatives, TBzTD/ZBEC/ZDEC, led to the fastest reduction in swelling, achieving the target crosslinking within 7 days. So, the curing time required to reach the 103% swelling index was reduced from 10 days (ZDEC and ZBEC) to 7 days. TBzTD and ZBEC binary and TBzTD alone were also effective, promoting sufficient crosslinking to reach the standard threshold (103% swelling) within 9 days.

Obtaining this 103% swelling index confirms the effectiveness of TBzTD/ZBEC/ZDEC as accelerators to reduce the curing time while maintaining the required crosslink density for product quality.

#### 3.1.2. FTIR Analysis of Accelerator Types in Sulfur Vulcanization of NRL Films

The consulted expert sources focused on the formation and mitigation of nitrosamines in natural rubber latex balloons. One primary source details research into substituting problematic vulcanization accelerators like ZDEC with safer alternatives such as ZBEC and TBzTD to eliminate the carcinogenic nitrosamine content while maintaining necessary mechanical properties like tensile strength and elasticity. The chemical and mechanical properties of the vulcanizate formed using a nitrosamine-free combination of 0.03 phr ZDEC with TBzTD/ZBEC 0.36/0.36 phr are compared to the properties of a vulcanizate with ZDEC in a neat recipe.

The experiment compared the use of two and three accelerators with the use of a single accelerator, specifically from ZBEC, TBzTD, and ZDEC. The NRL formulation uses a blend of two (TBzTD and ZBEC) or three types of accelerators with ZDEC added to enhance the accelerator system between TBzTD and ZBEC. The combinations of accelerators significantly improved the vulcanization efficiency, thermal stability, and mechanical properties, demonstrating the importance of synergistic accelerator selection for optimizing natural rubber balloons.

**Characterization by Fourier transform infrared spectroscopy**

**Figure 3:**
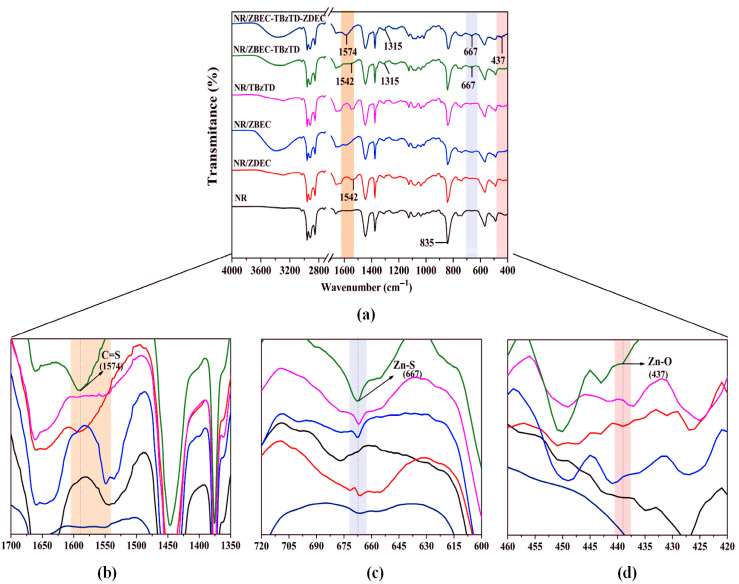
The FTIR spectra of NRL film formulations with different accelerators. (**a**) the FTIR spectra of the NRL; (**b**) the C=S band; (**c**) a cross-linking; (**d**) Zn–O bonding.

The NRL formulation characterization in Figure 3 shows the Fourier transform infrared (FTIR) analysis that informs us of the functional groups and the crosslinks formed.

Figure 3a presents the FTIR spectra of the NRL formulations, demonstrating that the mixed-accelerator system (ZBEC/TBzTD/ZDEC) produces stronger C=S, C–S, Zn–S, and Zn–O peaks than single-accelerator systems. Around 1315 cm^−1^, distinct C–S peaks indicate enhanced crosslink density, especially in formulations incorporating multiple accelerators [21]. In Figure 3b, in the 1574 cm^−1^ region, the appearance and growth of the C=S band [22,23], together with its larger integrated area, confirms additional reactions of the accelerator with vulcanizing agents, leading to modified sulfur-containing structures. The distinct peak near 667 cm^−1^ confirms the formation of Zn–S bonds in Figure 3c, a key cross-linking of rubber chains in the sulfur vulcanization process [24]. In Figure 3d, the band at approximately 437 cm^−1^ evidences Zn–O bonding [25].

The spectral evidence confirms that the synergistic accelerator system (ZBEC/TBzTD/ZDEC) in NRL facilitates more efficient sulfur crosslinking and the formation of complex Zn–S, Zn–O, and C=S bonding networks. This enhanced crosslink density strengthens the mechanical performance and thermal resistance, outperforming single or dual accelerator systems (Table 3). Importantly, the formulation achieves these improvements without any or at a low generation of nitrosamine compounds.

#### 3.1.3. Comparative Analysis of Mechanical Properties

The mechanical properties before and after accelerated aging in Table 3 indicate that changing the accelerator system greatly impacts the mechanical properties of natural rubber latex balloons. Formulations using single accelerators such as ZBEC, TBzTD or ZDEC provide moderate tensile strength and elongation at break, while binary combinations offer only limited improvements.

Notably, the ternary blend of 0.36 phr ZBEC/0.36 phr TBzTD/0.03 phr ZDEC, even with a minimal amount of ZDEC, achieved the highest tensile strength (22.13 ± 1.81 MPa) and elongation at break (~1153%) before aging, indicating a highly tough and well-developed crosslink network. The tensile strength of the optimized balloon compound remains within the internal specification range provided by the B.K. Latex Product Co., Ltd., with typical values around 25.792 MPa, elongation at break of approximately 1067%, and a 300% modulus of 1.569 MPa. In practice, the property that mainly influences whether a balloon is difficult to inflate is the 300% modulus, which should be relatively low to avoid excessive stiffness, while a sufficiently high tensile strength is still desirable to prevent rupture during inflation and use. This superior performance demonstrates a synergistic effect, where the combination of these three accelerators leads to more efficient vulcanization and greater reinforcement of the polymer structure. The ternary blend also resulted in the highest moduli at all elongation levels before aging, which is associated with increased initial stiffness and higher crosslink density in the rubber matrix. The thermal stability for natural rubber balloons, as described in standards such as TIS 685, BgVV, 2002 [26], and EN 71-12:2016 [12], is not defined by explicit numerical limits for thermal degradation, but instead is evaluated indirectly through compliance with mechanical and physical tests after heat aging. Thermal stability is necessary so that the balloons continue to meet the required mechanical safety criteria (e.g., flexibility, absence of brittleness, and retention of tensile performance within acceptable range) following the specified heat-aging conditions, to ensure that the produce remains mechanically reliable throughout storage and use. After accelerated aging, the tensile strength and elongation when using the ternary system implies improved thermal resistance compared to single or binary accelerator systems. Each type of accelerator or combination thereof influences both the mechanical and thermal properties, with the three-accelerator system consistently yielding the best results. Overall, the synergistic accelerator system of ZBEC, TBzTD and ZDEC provides a high-performance, strong, and stable NRL balloon product, surpassing formulations with only one or two accelerators. The benefits impact both the mechanical properties and service life.

#### 3.1.4. Thermal Stability of Sulfur Vulcanization

**Figure 4:**
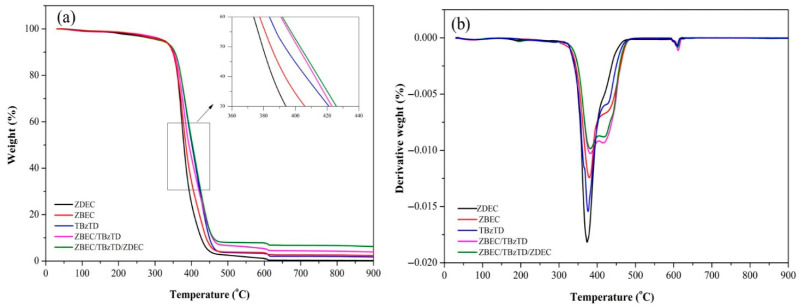
TGA (**a**) and DTG (**b**) graphs showing weight losses with the alternative types of accelerators in NRL formulation for balloons by sulfur vulcanization.

Figure 4 illustrates the thermal stability of NRL vulcanizates prepared with different accelerator systems, as evaluated by TGA and derivative thermogravimetry (DTG).

From Table 4, TGA curves in Figure 4a, and DTG in Figure 4b, it is found that all formulations exhibit a similar single-step degradation, with rapid weight loss between 180 and 320 °C being associated with the decomposition of the rubber backbone. The ZBEC/TBzTD/ZDEC formulation provides the greatest thermal stability, as evidenced by its highest T_onset_ (357.75 °C), T_max_ (384.15 °C), and final decomposition temperature (403.04 °C), together with the largest residual mass of 7.66%, indicating the presence of thermally stable species (e.g., ZnS, ZnO) within the measurement range. The DTG curves (Figure 4b) show the rate of that mass change and display a distinct shoulder at 400–500 °C arising from additional degradation of the crosslinked network and residue, confirming that the combined accelerator system enhances both the onset and completion temperatures of degradation compared with the other formulations.

This balance between enhanced thermal stability and sulfur crosslinking makes the novel formulations suitable for applications requiring both mechanical performance and heat resistance [27,28,29]. These results confirm an accelerator synergistic effect in NRL compound sulfur vulcanizates, with increased thermal stability while maintaining adequate sulfur crosslinking for NRL balloon applications.

From the resulting increases in the mechanical strength, elasticity, and modulus of the NR compound, the synergistic accelerator system (ZBEC/TBzTD/ZDEC) in sulfur vulcanization is confirmed.

**Figure 5:**
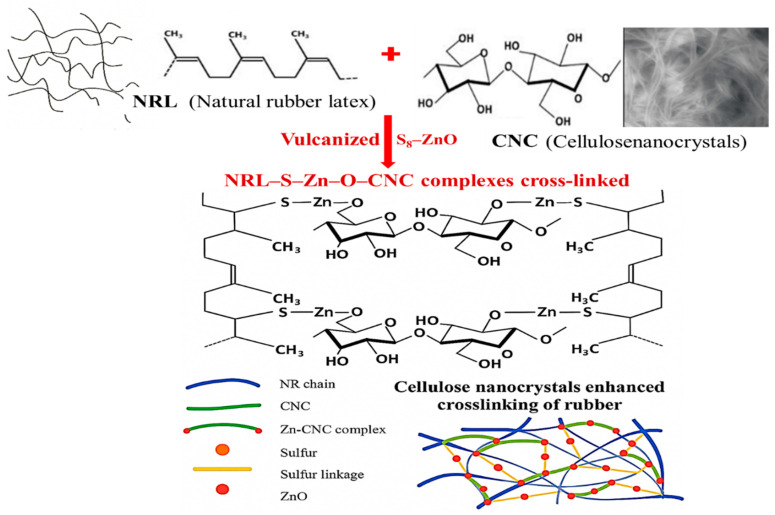
The formation of sulfur crosslinks in NRL–S_8_–Zn–O–CNC complexes.

Figure 5 illustrates the interactions and cross-linking process between natural rubber latex (NRL), sulfur, zinc oxide, and cellulose nanocrystals, enhancing the entanglement and interfacial network within the rubber matrix. The CNC not only improves the dispersion of zinc oxide but also participates in the formation of ionic Zn–O–CNC crosslinks, which further connect to rubber chains via sulfur bridges. This leads to a robust NRL–S_8_–Zn–O–CNC network, increasing the number of crosslink points and entanglement between rubber and filler.

The curing mechanism indeed involves the CNC. The role of CNC is now exposed by reports that cellulose-based fillers can participate in zinc-containing curing domains and influence vulcanization behavior, which supports the plausibility of interactions between CNC surface groups, ZnO, and the sulfur–accelerator system. Regarding the proposed coupling of cellulose to the polymer via a Zn–S bridge, this structure is hypothetical and consistent with the generally accepted view that the active Zn complexes form at, or in association with, ZnO-containing domains. Additionally, we note that (i) Zn–accelerator–sulfur complexes generated at the ZnO surface can migrate into the rubber matrix, (ii) hydroxyl and modified functional groups on CNC can coordinate or interact with zinc species, and (iii) such interactions may contribute to a combined filler–curing network, rather than a single, unique structure. The proposed structures are one plausible realization among several possibilities, supported indirectly by the curing behavior and mechanical property trends, rather than by direct structural evidence. For related details and discussion, we refer to the works of Low et al. (2021) [30], Blanchard et al. (2020) [31], and Suchat et al. (2024, 2025) [13,32].

#### 3.1.5. Nitrosamine Testing

The results of nitrosamine testing in the NRL formulation for balloons (Table 5), using GC-TEA according to EN 12868:2017, confirm that the synergistic activities of the novel combinations 0.03 phr ZDEC; 0.36 phr TBzTD/0.36 phr ZBEC and TBzTD, ZBEC, ZDEC, TBzTD/ZBEC, could be effective accelerator systems to replace the unsafe ZDEC neat for the sulfur vulcanization of NRL balloons.

**Figure 6:**
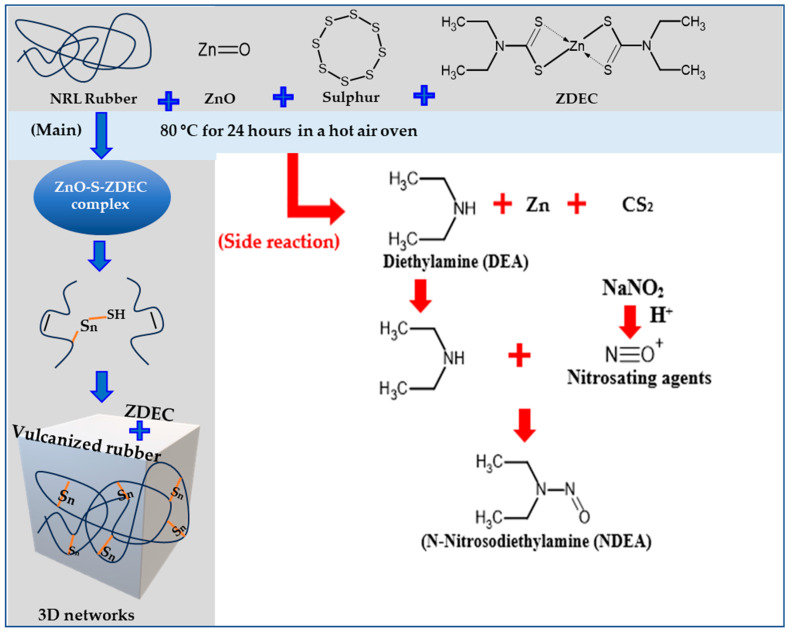
Diagram illustrating the reaction mechanisms that lead to the formation of nitrosodiethylamine in NRL formulation for balloons with ZDEC accelerator.

Table 5 indicates that migratable levels of nitrosamines were found: in particular, the detected levels of *N*-nitrosodimethylamine (NDMA) and *N*-nitrosodiethylamine (NDEA) were 0.13 and 0.10 mg/kg, respectively, in the NRL formulation for balloons using zinc diethyldithiocarbamate (ZDEC). The nitrosamine is a compound formed from the precursor diethylamine, which results from the decomposition of the accelerator ZDEC during the vulcanization. ZDEC is classified as a highly effective “super-accelerator” for NR latex but is also considered “unsafe” and is one of the “primary problematic accelerators” because it can form carcinogenic nitrosamines. The sources include diagrams showing the reaction mechanism in Figure 6. NDEA substance is classified by the International Agency for Research on Cancer (IARC) as a Group 2A carcinogen (“probably carcinogenic to humans”) and is considered a very potent carcinogen [26].

Figure 6 illustrates the mechanisms of NDEA formation from ZDEC that involve three steps: (1) decomposition of ZDEC during vulcanization, releasing diethylamine, a secondary amine; (2) nitrosation reaction where diethylamine reacts with nitrosating agents such as nitrites or nitrogen oxides, which may be present during the manufacturing process or in the environment; and (3) formation of NDEA, a nitrosamine classified as a carcinogen. The inductive and resonance effects of the amine group, along with steric factors, influence the formation of zinc–accelerator complexes.

The meaning of the red side-reaction arrow is to indicate that the chemical reaction leading to the release of *N*-nitrosodiethylamine (NDEA) does not occur in the developed formulation. The nitrosation pathway is suppressed by the novel accelerator system. The absence of NDEA formation has been verified through expert chemical review and confirmed by analytical testing, which showed non-detectable (nd) levels below the reporting limit. Regarding the mechanism, a conventional accelerator complex such as ZDEC can release NDEA because it decomposes during the heating for vulcanization, producing secondary amines (specifically diethylamine). These secondary amines then combine with nitrosating agents (such as nitrogen oxides (NOx) from the atmosphere or sodium nitrite (NaNO_2_) used in processing) to form the carcinogenic *N*-Nitrosodiethylamine (NDEA). The diagram aims to demonstrate that while conventional rubber compounds are prone to nitrosamine formation, the developed “nitrosamine-safe” formulation (using tetrabenzylthiuram disulfide (TBzTD) and zinc n-butylbenzothiazole-2-sulfenamide (ZBEC) as a big group) employs chemical structures that do not form secondary amines. This substitution effectively prevents the generation of regulated nitrosamines while maintaining the required mechanical properties for balloon production. While having chemical plausibility and consistency with established mechanistic understanding, the proposed reaction pathways remain indirectly supported by the works of Low et al. (2021) [30], Blanchard et al. (2020) [31] and Suchat et al. (2025) [32].

For safety reasons, we recommend choosing safer alternative accelerators, such as ZBEC and TBzTD, to replace ZDEC in the production of NRL products, especially those that come into contact with humans, such as balloons. The focus on the alternative accelerator compounds fulfilling the requirement of releasing no carcinogenic nitrosamines ensures that these alternative systems meet both the safety criterion of non-detectable carcinogenic nitrosamines and the required performance for balloon production, thereby strengthening the central message and focus of this study.

**Figure 7:**
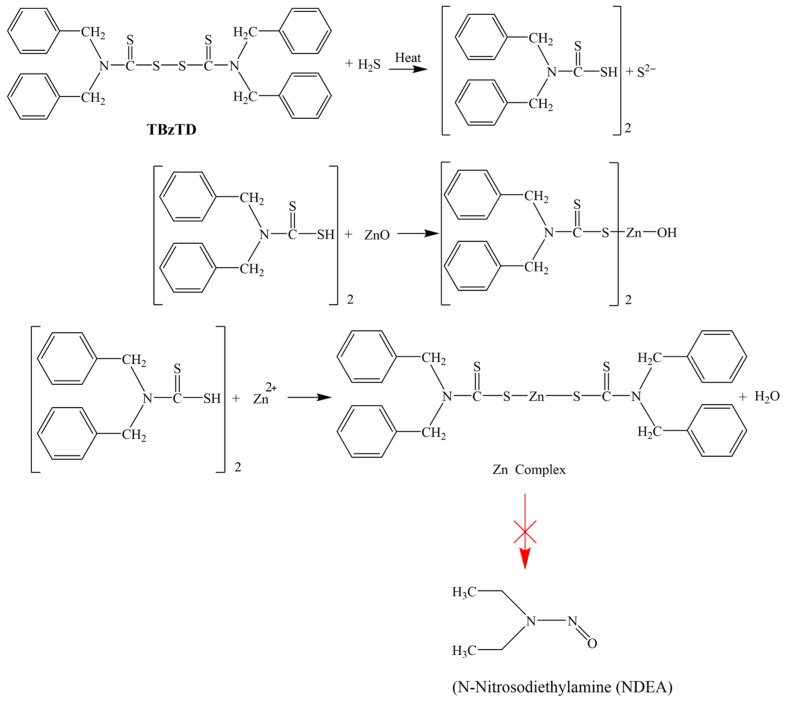
Diagram of the reaction mechanisms in the nitrosamine-free NRL formulation with TBzTD.

**Figure 8:**
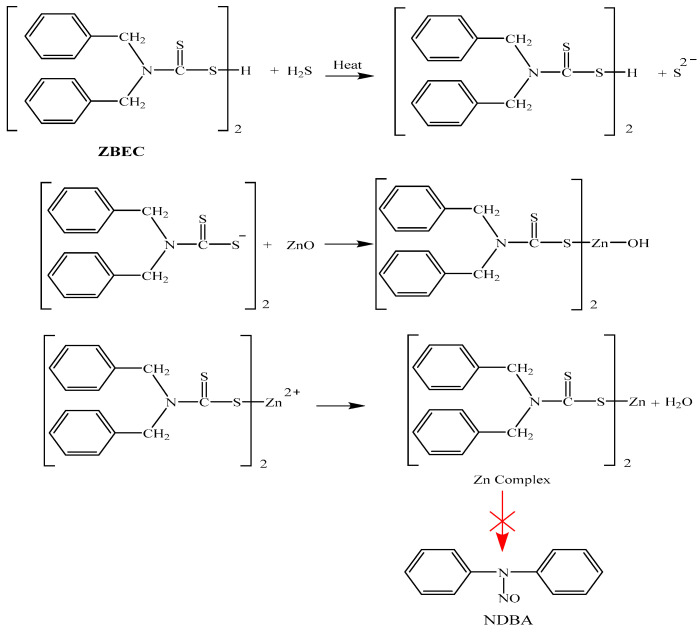
The reaction mechanisms in the nitrosamine-free NRL formulation for balloons with ZBEC.

The use of multiple accelerants, whether two or three, compared to a single accelerant like TBzTD, still maintains the nitrosamines values below the latex balloon standard limits, as shown in Table 5 and Figure 7 and Figure 8. The tables and figures corroborate that the novel formulations are safe for balloons made from natural rubber latex (NRL) [32,33].

Tetrabenzyl thiuram disulfide (TBzTD), with the chemical formula C30H28S4N2 and a molecular weight of 542.78, undergoes a reaction, shown in Figure 7. The induction of the amine group, along with steric factors, influences the formation of zinc–accelerator complexes. Nonetheless, as shown in Table 5, *N*-nitrosodibenzylamine (NDBzA) is slightly produced, but this is safe and not a carcinogenic nitrosamine.

Based on the data in Table 5 and Figure 8, when Zinc dibutyldithiocarbamate (ZBEC) is used in an NRL formulation for balloons, NDMA and NDEA are not detected (reporting limit 0.01 mg/kg). The inductive effect of the amine group, along with the steric factors, plays a significant role in influencing the formation of zinc complexes and accelerators during vulcanization. These factors affect how compounds interact and bond, thereby impacting the vulcanization. Comparative testing of NRL compounds showed clearly that the ZBEC accelerator contributed to sulfur vulcanization, and if a nitrosation reaction occurs, the resulting nitrosamine will be NDBA (nitrosodibutylamine) at a low concentration, which is safe and not a carcinogenic nitrosamine (Table 5).

It is also noteworthy that even though ZBEC could theoretically form NDBA, the test results shown in Table 4 have some NDBA, which is safe and not carcinogenic [19]. Using a ZBEC accelerator is an effective method for avoiding the formation of NDMA and NDEA in products, which is a key reason for its selection in formulations requiring safety by not carrying carcinogenic nitrosamines.

According to Table 5, the investigation of a novel synergistic accelerator system comprising a little bit of ZDEC (0.03 phr) with the combination of TBzTD (0.36 phr), and ZBEC (0.36 phr), suggests that this combination could be an effective and safer alternative to the unregulated ZDEC for the sulfur vulcanization of NRL balloons.

The formula of TBzTD/ZBEC and ZDEC in the NRL compound for balloons, incorporating the accelerators that complied with established nitrosamine and nitrosatable substances safety standards, appears to be suitable as a safer alternative to conventional accelerators. The bulky phenyl substituents exert steric hindrances that decrease the alkalinity of the amine. This reduction in alkalinity enhances the stability of the zinc–accelerator complex, which in turn suppresses the release of the reactive intermediates that are responsible for nitrosamine generation, thereby effectively reducing nitrosamine formation.

While TBzTD or ZBEC was specifically developed as a safer alternative to traditional, problematic accelerators like ZDEC, it cannot be considered completely safe. Here, the balloon formulation was designed to have an effective accelerator system to replace the use of unsafe ZDEC neat.

Using a combination of TBzTD and ZBEC accelerators, supplemented with a very small amount (0.03 phr) of ZDEC, is a highly effective strategy for natural rubber latex balloon formulations. This approach achieves two critical goals.

(1) Enhanced product properties. Through a synergistic effect, it produces balloons with significantly higher tensile strength and thermal stability compared to other accelerator systems.

(2) High safety level. By relying on primarily safe accelerators and minimizing the precursor for harmful nitrosamines, it yields a final product with lower levels than the standard limits, thereby meeting the strict safety standards for rubber products that may be used by children.

**Figure 9:**
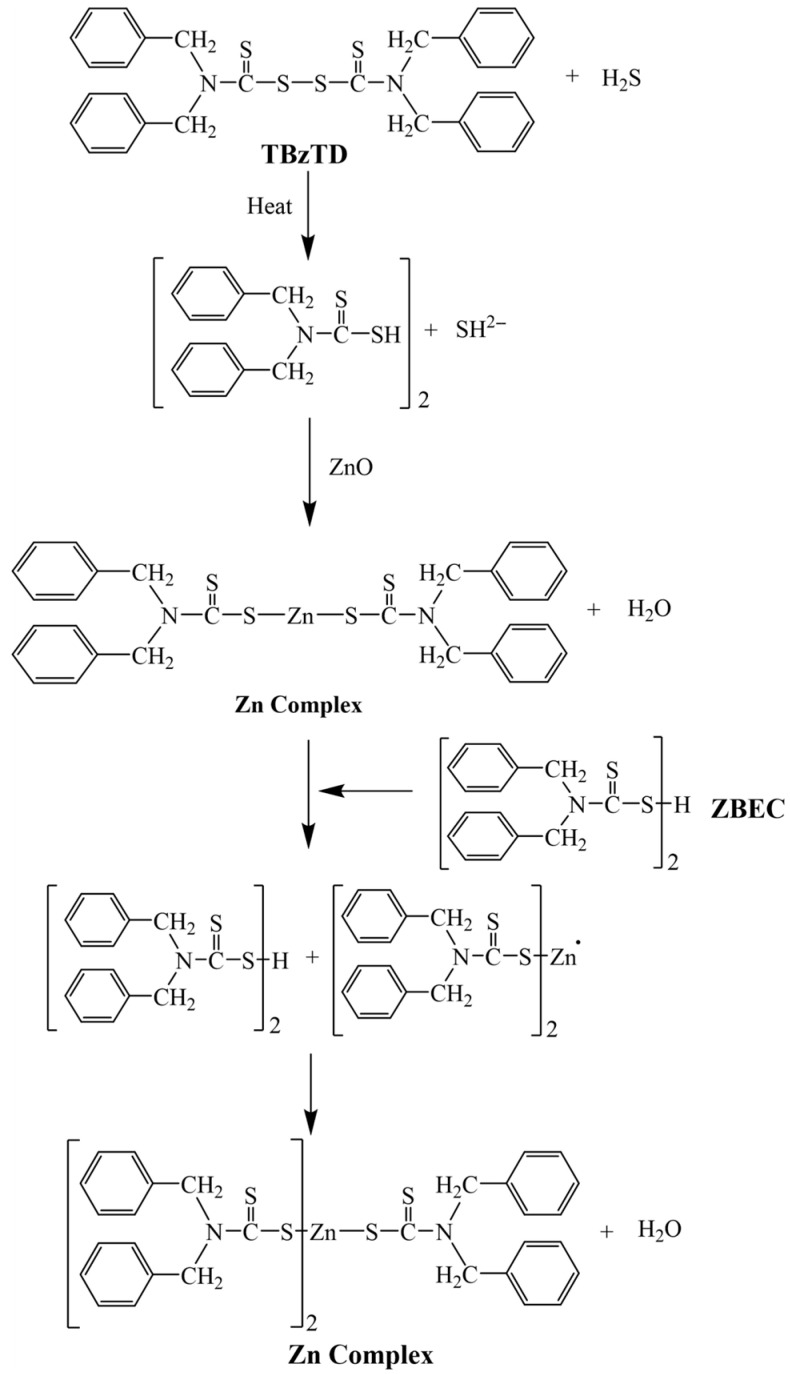
Reaction mechanisms in NRL formulation for balloons, based on a nitrosamine-free TBzTD/ZBEC combination.

The reaction mechanisms in nitrosamine-free NRL formulation for balloons with a synergistic accelerator combination of TBzTD and ZBEC are shown in Figure 9. The nitrosamine testing for the NRL balloon compounds that used the three alternative accelerator combinations, TBzTD/ZBEC/ZDEC, was done using GC-TEA according to EN 12868:2017 [15] and BgVV (2002) [26]. It confirmed that the total migration values of the nitrosamine and the nitrosatable substances were below the standard balloon limits (0.05 mg/kg for nitrosamines and 1.0 mg/kg for nitrosatable substances). Based on discussions with balloon manufacturers, exporters, and experts in this field, the proposed approach is considered acceptable, ensuring no exposure to carcinogenic nitrosamines for humans.

## 4. Conclusions

This study developed a sulfur-vulcanized NRL compound formulation for balloon products, exceeding the required mechanical properties while controlling the carcinogenic nitrosamine content for compliance with the stringent safety standards. In experiments, the types and amounts of accelerators were manipulated, while controlling for the 103% swelling index and 62% total solid content targets. It was found that the synergistic accelerator system of TBzTD (0.36 phr) and ZBEC (0.36 phr), with a little bit of ZDEC (0.03 phr) gave significantly improved mechanical performance without detectable carcinogenic nitrosamines, outperforming formulations using a combination of TBzTD and ZBEC or each accelerator alone. Mechanical testing revealed the highest tensile strength (22.1 ± 1.8 MPa), elongation (1153 ± 78%), and 300% modulus (1.30 ± 0.07 MPa), complying with balloon product standards. The thermal stability was confirmed by its good mechanical properties after accelerated aging, and by thermogravimetric analysis, which showed a maximum decomposition temperature of 384.15 °C. Overall, the synergistic use accelerators enhanced the vulcanization efficiency, thermal stability, and mechanical properties, highlighting the critical role of accelerator synergy in optimizing natural rubber balloon performance, with safety standards concerning carcinogenic nitrosamine content.

## Figures and Tables

**Table 1:** The compound formulations of NRL used in the present study.

Ingredient in Formulation	Content (phr ^a^)					Function
	1	2	3	4	5	
60% HA-NR latex	100	100	100	100	100	Backbone (main component)
10% Potassium hydroxide	1.0	1.0	1.0	1.0	1.0	Stabilizers 1
10% Potassium oleate	1.0	1.0	1.0	1.0	1.0	Stabilizers 2
50% Sulfur	1.5	1.5	1.5	1.5	1.5	Vulcanizing Agent
50% ZnO	1.5	1.5	1.5	1.5	1.5	Activator
5% CNC	2.0	2.0	2.0	2.0	2.0	Filler
50% Wingstay L	1.5	1.5	1.5	1.5	1.5	Polyphenol Antioxidant
50% Accelerators ^b^	0.75	0.75	0.75	0.75	0.75	Accelerators

Note: ^a^ Parts per hundred rubber (phr) and ^b^ 50% accelerators in Table 2.

**Table 2:** The accelerator content in NRL compound formulations.

Formula No. #	Accelerator Type	Content (phr)			
		TBzTD	ZBEC	ZDEC	Total
1	ZDEC	0.75	-	-	0.75
2	ZBEC	-	0.75	-	0.75
3	TBzTD	-	-	0.75	0.75
4	ZBEC/TBzTD	0.375	0.375	-	0.75
5	ZBEC/TBzTD/ZDEC	0.36	0.36	0.03	0.75

**Table 3:** Mechanical properties of NRL formulation for balloons prepared with alternative accelerators.

Type of Accelerator	Mechanical Properties				
	Tensile Strength (MPa)	Elongation at Break (%)	100% Modulus (MPa)	200% Modulus (MPa)	300% Modulus (MPa)
**Before aging**					
ZBEC	18.74 ± 0.90	1079.31 ± 83	0.54 ± 0.01	0.81 ± 0.01	1.08 ± 0.03
TBzTD	19.57 ± 1.47	1136.74 ± 71	0.46 ± 0.01	0.65 ± 0.08	0.83 ± 0.03
ZDEC	21.36 ± 1.10	1040.86 ± 60	0.55 ± 0.07	0.88 ± 0.12	1.20 ± 0.14
ZBEC/TBzTD	17.91 ± 0.75	1115.82 ± 55	0.50 ± 0.13	0.67 ± 0.05	0.84 ± 0.10
ZBEC/TBzTD/ZDEC	22.13 ± 1.81	1152.90 ± 78	0.69 ± 0.02	0.95 ± 0.04	1.30 ± 0.07
**After aging**					
ZBEC	12.10 ± 2.11	626.50 ± 34	1.06 ± 0.01	1.81 ± 0.02	3.30 ± 0.02
TBzTD	13.25 ± 1.72	736.85 ± 26	1.24 ± 0.07	2.39 ± 0.12	3.21 ± 0.15
ZDEC	15.50 ± 0.89	851.9 ± 35	1.53 ± 0.02	2.00 ± 0.01	4.59 ± 0.10
ZBEC/TBzTD	13.85 ± 0.70	785.98 ± 15	0.95 ± 0.03	1.17 ± 0.03	3.50 ± 0.04
ZBEC/TBzTD/ZDEC	14.23 ± 1.81	892.91 ± 8	1.69 ± 0.02	2.48 ± 0.04	4.04 ± 0.07

**Table 4:** Thermal stability analysis of NRL film formulations with alternative accelerators for sulfur vulcanization.

Rubber Sample Type	T_onset_ * (°C)	T_max_ ** (°C)	Final Decomposition Temperature (FDT)	Final Residue (%)
ZBEC	356.01	374.78	379.79	0.38
TBzTD	356.13	378.01	391.14	4.47
ZDEC	358.05	376.47	384.47	2.63
ZBEC/TBzTD	342.16	382.62	400.56	2.05
ZBEC/TBzTD/ZDEC	357.75	384.15	403.04	7.66

**Note**: * T_onset_ is the decomposition onset temperature, T_max_ ** is the temperature at the maximum decomposition rate.

**Table 5:** Test of migratable nitrosamines in NRL formulations for balloons.

Test Parameter	1-ZDEC		2-ZBEC		3-TBzTD		4-ZBEC + TBzTD		5-ZBEC + TBzTD + ZDEC	
	Nitrosamines	Nitrosatable Substances	Nitrosamines	Nitrosatable Substances	Nitrosamines	Nitrosatable Substances	Nitrosamines	Nitrosatable Substances	Nitrosamines	Nitrosatable Substances
	(mg/kg)	(mg/kg)	(mg/kg)	(mg/kg)	(mg/kg)	(mg/kg)	(mg/kg)	(mg/kg)	(mg/kg)	(mg/kg)
1. NDMA	0.130	0.861	nd	nd	0.028	0.495	0.030	0.480	0.034	0.565
2. NDEA	0.100	0.405	nd	nd	nd	0.372	nd	0.250	0.006	0.329
3. NDPA	nd	nd	nd	nd	nd	nd	nd	nd	nd	nd
4. NDiBA	nd	nd	nd	nd	nd	nd	nd	nd	nd	nd
5. NDBA	nd	nd	0.045	0.300	nd	0.040	0.010	0.220	0.004	0.060
6. NPIP	nd	nd	nd	nd	nd	nd	nd	nd	nd	nd
7. NPYR	nd	nd	nd	nd	nd	nd	nd	nd	nd	nd
8. NMOR	nd	nd	nd	nd	nd	nd	nd	nd	nd	nd
9. NEPhA	nd	nd	nd	nd	nd	nd	nd	nd	nd	nd
10. NMPhA	nd	nd	nd	nd	nd	nd	nd	nd	nd	nd
11. NDiNA	nd	nd	nd	nd	nd	nd	nd	nd	nd	nd
12. NDBzA	nd	nd	nd	nd	0.015	0.076	0.004	0.035	0.004	0.040
Total	0.230	1.266	0.045	0.300	0.043	0.983	0.044	0.985	0.048	0.994
	Fail	Fail	pass *	pass **	pass *	pass **	pass *	pass **	pass *	pass **

Note: nd, not detected or lower than the reporting limit (* migratable nitrosamines, 0.05 mg/kg, and ** migratable nitrosatable substances 1.0 mg/kg). Requirements according to results of nitrosamine testing in the NRL formulation for balloons, using GC-TEA according to EN 12868:2017.

## Data Availability

The original contributions presented in this study are included in the article. Further inquiries can be directed to the corresponding author.
